# Evaluation of Antitumor Activity of Xanthones Conjugated with Amino Acids

**DOI:** 10.3390/ijms25042121

**Published:** 2024-02-09

**Authors:** Flávia Barbosa, Joana Araújo, Virgínia M. F. Gonçalves, Andreia Palmeira, Andrea Cunha, Patrícia M. A. Silva, Carla Fernandes, Madalena Pinto, Hassan Bousbaa, Odília Queirós, Maria Elizabeth Tiritan

**Affiliations:** 1 UNIPRO—Oral Pathology and Rehabilitation Research Unit, University Institute of Health Sciences (IUCS-CESPU), 4585-116 Gandra, Portugal; a24728@alunos.cespu.pt (F.B.); virginia.goncalves@cespu.pt (V.M.F.G.); andrea.cunha@iucs.cespu.pt (A.C.); patricia.silva@cespu.pt (P.M.A.S.); hassan.bousbaa@iucs.cespu.pt (H.B.); odilia.queiros@iucs.cespu.pt (O.Q.); 2 Laboratory of Organic and Pharmaceutical Chemistry, Department of Chemical Sciences, Faculty of Pharmacy, University of Porto, Rua Jorge Viterbo Ferreira, 228, 4050-313 Porto, Portugal; m.araujo.joana@gmail.com (J.A.); apalmeira@ff.up.pt (A.P.); cfernandes@ff.up.pt (C.F.); madalenakijjoa@gmail.com (M.P.); 3 1H-TOXRUN—One Health Toxicology Research Unit, University Institute of Health Sciences (IUCS), University Institute of Health Sciences-CESPU (IUCS-CESPU), 4585-116 Gandra, Portugal; 4 CIIMAR-Interdisciplinary Center for Marine and Environmental Research, University of Porto, Avenida General Norton de Matos, 4450-208 Matosinhos, Portugal

**Keywords:** chiral derivatives of xanthones, cancer, glycoprotein-P, multidrug resistance

## Abstract

Cancer is a complex disease characterized by several alterations, which confer, to the cells, the capacity to proliferate uncontrollably and to resist cellular death. Multiresistance to conventional chemotherapy drugs is often the cause of treatment failure; thus, the search for natural products or their derivatives with therapeutic action is essential. Chiral derivatives of xanthones (CDXs) have shown potential inhibitory activity against the growth of some human tumor cell lines. This work reports the screening of a library of CDXs, through viability assays, in different cancer cell lines: A375-C5, MCF-7, NCI-H460, and HCT-15. CDXs’ effect was analyzed based on several parameters of cancer cells, and it was also verified if these compounds were substrates of glycoprotein-P (Pgp), one of the main mechanisms of resistance in cancer therapy. Pgp expression was evaluated in all cell lines, but no expression was observed, except for HCT-15. Also, when a humanized yeast expressing the human gene MDR1 was used, no conclusions could be drawn about CDXs as Pgp substrates. The selected CDXs did not induce significant differences in the metabolic parameters analyzed. These results show that some CDXs present promising antitumor activity, but other mechanisms should be triggered by these compounds.

## 1. Introduction

Cancer is considered the first or second leading cause of death before the age of 70 in economically developed and developing countries, respectively [1], being characterized, among other characteristics, by uncontrolled cell proliferation and resistance to cell death. Cancer chemotherapeutic drugs are particularly toxic to cancer cells; however, they are often non-specific, being responsible for the important side effects associated with cancer treatment [2]. Moreover, many cancers often develop resistance to several antitumor drugs, even with different structures and mechanisms of action, which is one of the major obstacles to treatment success. The screening of new potential antitumor drugs that are effective and more specific and that can overcome the phenotype of multidrug resistance is then of major importance in the field of cancer therapy. The use of scaffolds for drug discovery allows for the exploration of different chemicals, providing structural templates to optimize molecules with potential anticancer activity. For example, heterocyclic building blocks, like thiophenes, pirrole, or pyrazoles, demonstrated high efficiency in the treatment of different types of cancer, including metastatic models [3,4,5,6]. Over eighty-five percent of FDA-approved molecules contain heterocycles and have indicated potential benefits against a range of cancer treatments [7]. These compounds can also possess chirality, increasing the chemical space and consequently the number of derivatives that can have pharmacologic potential [7]. In fact, recent advances in this field allowed for the identification of several compounds with different pharmacologic activities, including anticancer. A xanthone scaffold is also a heterocyclic structure and has attracted attention in recent anticancer research [8]. Nature is the main source of chiral bioactive compounds, which can be used as therapeutic agents, such as xanthone derivatives. Many naturally occurring xanthones, isolated from plants and marine sources, are chiral and exhibit interesting biological activities [8,9,10,11]. Nevertheless, one of the main biological activities reported within this class of compounds is their antitumor activity [12,13,14]. Xanthone is an aromatic oxygenated heterocyclic molecule, with a dibenzo-γ-pirone scaffold, known as 9*H*-xanthen-9-one, which permits chemical modifications in different positions of the molecule scaffold, including the association of the chiral moiety, allowing for the preparation of libraries of derivatives for enantioselective structure–activity relationships [14,15]. The rule of stereochemistry in the biological activity of natural or synthetic compounds has been demonstrated over the years and can be considered one of the main roads for the discovery and development of new drugs [8]. The enantiomers generally have different pharmacokinetic and/or pharmacodynamic properties, and the use of single enantiomers has often demonstrated advantages in terms of efficacy, potency, selectivity, and safety [15,16,17,18]. Thus, the enantioselectivity is recommended to be evaluated in the early stage of drug development [18,19].

The xanthone nucleus is considered a privileged structure, given its ability to have diverse substituents and, consequently, bind to several biotargets [12], and its association with a chiral moiety can improve the potency and the selectivity of their derivatives in the biological activity [11,15,20,21]. The introduction of amino acids into natural products is expected to improve both pharmacodynamics and pharmacokinetics of these compounds and minimize their adverse effects [22]. The association of natural compounds with amino acids for therapeutic applications is an approach that offers a potentially effective system of drug discovery that can permit the development of pharmacologically active and pharmacokinetically acceptable molecules. Several examples of natural compounds conjugated with amino acids have enhanced pharmacokinetic characteristics, including better absorption and distribution properties, reduced toxicity, and increased physiological effects [23]. The association of xanthone with amino acids as potential anticancer and DNA-binding agents has been reported, but the enantioselectivity was not explored [24]. Recently, we reported the synthesis of a library of sixty chiral derivatives of xanthones (CDXs) based on coupling a carboxyxanthone with amino esters and amino acids following the chiral pool strategy [15]. The cytocompatibility and anti-inflammatory activity were studied for forty-four of the newly synthesized CDXs. The levels of a proinflammatory cytokine targeted in the treatment of several inflammatory diseases were significantly decreased in the presence of many CDXs. Enantioselectivity could be observed for the majority of the evaluated enantiomeric pairs [15]. Here, the aim of this study was to screen a library of previously synthesized CDXs in a panel of cancer cell lines to identify the most promising compounds for further study as possible chemotherapy drugs, as well as to analyze their effect on parameters, like the mechanism of cell death, cell cycle, and metabolism, and verify whether the compounds under study were substrates of Pgp, one of the main mechanisms of resistance in cancer therapy.

## 2. Results and Discussion

### 2.1. Effect of Chiral Derivatives of Xanthones on the Growth of Human Tumor and Non-Tumor Cell Lines

**Figure 1:**
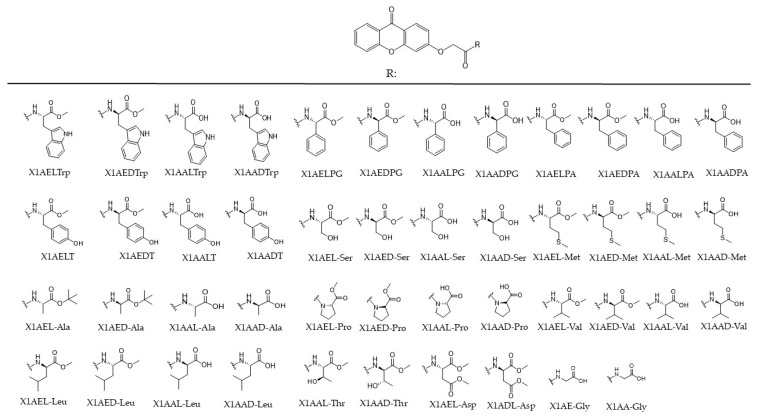
Library of synthesized CDXs. R corresponds to the substituent conjugated with the xanthone scaffold. X1—xanthone; AE—amino ester; AA—amino acid; the letters D and L correspond to the configuration of the enantiomers, respectively. The amino ester and acids used in conjugation were: Trp—tryptophan, PG—phenylglycine, PA—phenylalanine, T—tyrosine, Ser—serine, Met—methionine, Ala—alanine, Pro—proline, Val—valine, Leu—leucine, Thr—threonine, Asp—aspartic acid, Gly—glycine.

Tumor cells present characteristic alterations that contribute to their ability to survive and proliferate, which together are named the hallmarks of cancer, described in 2000 by Hanahan and Weinberg and meanwhile updated with four more hallmarks and four enabling characteristics [25,26,27]. These hallmarks give the cells the ability to proliferate, invade and metastasize, being also involved in resistance to therapy, one of the major causes of cancer cure failure. Tumor cells have several types of resistance mechanisms, including the expression of efflux pumps, like ABC transporters, with Pgp overexpression being one of the most common causes, which leads to the efflux of compounds that are their substrates, preventing their effect inside the cell [28,29]. Finding selective drugs with low toxicity to normal cells that are not Pgp substrates may thus be one of the ways to effectively treat this disease. Several studies concerning the evaluation of the biologic activity of xanthones demonstrated that they often present anti-tumor activity [13,30,31,32], besides many other interesting biological activities, including anti-oxidant, anti-inflammatory, anti-allergy, anti-bacterial, anti-fungal, and anti-viral activities [12,33,34]. Some cell targets for xanthone derivatives have already been described and include, among others, cyclin dependent kinases, important in cell cycle control [35,36], cytokines, kinases that modulate the activity of PI3K, Akt, and TOR proteins or proteins involved in apoptosis, like Bax, Bcl-2, or caspase 3 [37]. Nevertheless, their upshot on the activity of other hallmarks of cancer, like altered metabolism, and the enantioselectivity regarding chiral derivatives in anti-tumor activity have been poorly exploited. Hence, given their potential inhibitory activity on the growth of human tumor cells [8,32], as well as the possible enantioselectivity regarding chiral derivatives [14,20,21], the cytotoxic activity of forty-six previously synthesized CDXs (Figure 1) was evaluated in this work using a panel of tumor cell lines, including A375-C5 derived from melanoma, MCF-7 derived from breast adenocarcinoma, and NCI-H460 derived from non-small cell lung adenocarcinoma. These cells lines were chosen as cancer models as they are representative of common cancer types in humans (melanoma, breast cancer, and lung cancer) and were the cancer models used in the first assays of drug screening by the National Cancer Institute, USA, in drug screening platforms, as they are among the most sensitive to drug treatment [38]. Additionally, along with their clinical significance and the availability of prior data, these three cancer cell lines are widely recognized for their utility as experimental models in cancer research, particularly within our research group, ensuring the reliability and comparability of our results [14,20]. Concerning cytotoxicity, the specificity, the mechanism of cell death, effect on cell metabolism, and influence of Pgp on the action of the CDXs with the highest cytotoxic activity were further investigated. 

**Figure 2:**
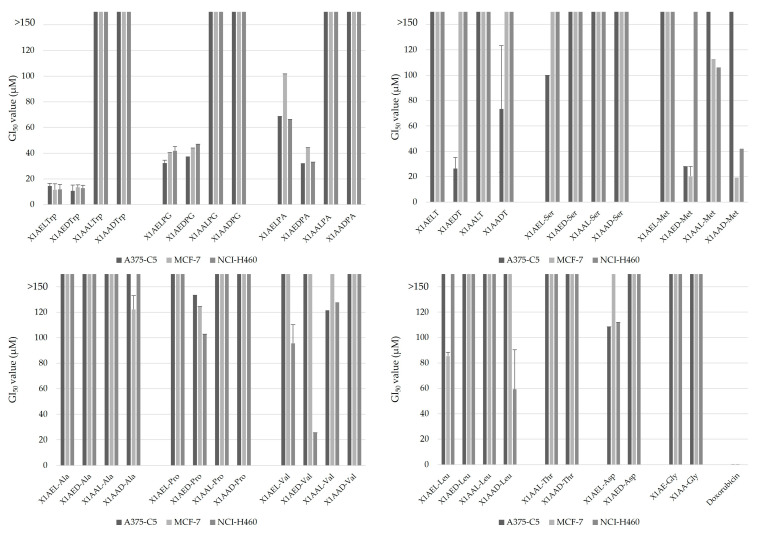
Effect of chiral derivatives of xanthones (CDXs) on tumor cancer cell growth, evaluated based on the respective GI_50_ value (in µM). The cell lines used in this assay were A375-C5 (melanoma), MCF-7 (breast cancer), and NCI-H460 (non-small lung cancer). The amino acids used in conjugation were as follows: Asp—aspartatic acid, Ala-alanine, Gly—glycine, Leu—leucine, Met—methionine, PA—phenylalanine, PG—phenylglycine, Ser—serine, Val—valine, Pro—proline, Thr—threonine, T—tyrosine, Trp—tryptophan.

The growth-inhibitory effect on the different cell lines, given by the GI_50_ (drug concentration that reduces total cell growth by 50%), evaluated via the SRB assay, are shown in Figure 2, and Table S1 in the Supplementary Material (SM). Screening results showed that most CDXs assayed presented high GI_50_ values, above 150 µM, the maximum value established to select compounds to proceed with further studies. However, many derivatives presented GI_50_ values lower than 50 µM, for at least two of the cell lines assayed (Table 1). In general, the CDXs associated with amino esters presented better cytotoxicity than their association with amino acids, except for D-methionine derivative (**X1AAD-Met**), which presented a GI_50_ = 19.33 ± 7.75 and GI_50_ = 42.1 ± 35.1 for MCF-7 and NCI-H460 cell lines, respectively. Comparing the cytotoxicity of the aliphatic and aromatic amino ester derivatives, the best results were correlated with the aromatic derivatives (Figure 2). For instance, the enantiomeric pair of the methyl ester of tryptophan (**X1AELTrp**/**X1AEDTrp**), phenylglycine (**X1AELPG**/**X1AEDPG**), and the enantiomer D of the methyl ester of phenylalanine (**X1AEDPA**) presented GI_50_ values lower than 50 µM, for all assayed cell lines. The derivative of the methyl ester of tyrosine (**X1AEDT**) also presented GI_50_ values lower than 50 µM for the cell line A375-C5 (GI_50_ = 26.52 ± 8.71). Regarding the aliphatic derivatives, only the amino ester derivative of D-methionine (**X1AED-Met**) and its amino acid derivative (**X1AAD-Met**) presented GI_50_ values lower than 50 µM for two of the assayed cell lines, while the methyl ester of the D-valine (**X1AED-Val**) has shown a GI_50_ = 25.6 ± 7.19 for the cell line NCI-H460. The results also showed that the cytotoxic activity of CDXs was often enantiomer-dependent (enantioselective), with the association with D enantiomers often being more cytotoxic (Figure 2, Table 1). The main evidence of enantioselectivity was observed with the derivatives of the methyl ester of D-methionine (**X1AED-Met**), presenting a considerable growth-inhibitory effect on two of the cell lines assayed, in opposition to what was observed for the L derivative, where GI_50_ values were higher than 100 µM. The enantioselectivity was also evident with the amino ester derivatives of tyrosine regarding the cytotoxicity against the cell line A375-C5, with the D amino ester derivative (**X1AEDT**) having a GI_50_ = 26.52 ± 8.71 and the L derivative (X1AELT) having a GI_50_ higher than 150 µM. Additionally, the amino ester derivative of methyl ester D-phenylalanine (**X1AEDPA**) presented lower values of GI_50_ than its L-enantiomer for all cell lines assayed. Nevertheless, this difference was not present for all the pairs and differed between the cell lines. From all the analyzed CDXs, the enantiomeric pair of the methyl ester derivatives of tryptophan (**X1AELTrp** and **X1AEDTrp**) were the ones that had the best anticancer activity, with the lowest values of GI_50_ for all assayed cell lines. Thus, these derivatives were selected for further studies, concerning their mechanism of action.

The selectivity index of the enantiomeric pair **X1AELTrp** and **X1AEDTrp** was evaluated. Interestingly, the GI_50_ values of **X1AELTrp** and **X1AEDTrp** in non-tumor HPAEpiC cells were 40.90 ± 1.17 and 43.58 ± 3.32 µM (Table 2), respectively, suggesting more prominent cytotoxic activity in tumor cells than in non-tumor cells (a greater than four-fold increase). Often, the selectivity index, a simple ratio of the GI_50_ calculated for healthy and tumor cells, has been used to show the selective cytotoxicity of different drugs [39]. Values of the selectivity index higher than 1 are indicative of desirable selectivity against cancer cells. Therefore, by calculating the selectivity index, we found remarkable degrees of selectivity, ranging from 2.82 to 4.09 (Table 2), for both compounds in all cell lines analyzed, highlighting the potential of **X1AELTrp** and **X1AEDTrp** as antitumor agents.

### 2.2. Pgp Expression in the Tumor Cell Lines A375-C5, MCF-7, NCI-H460, Caco-2, and HCT-15

**Figure 3:**
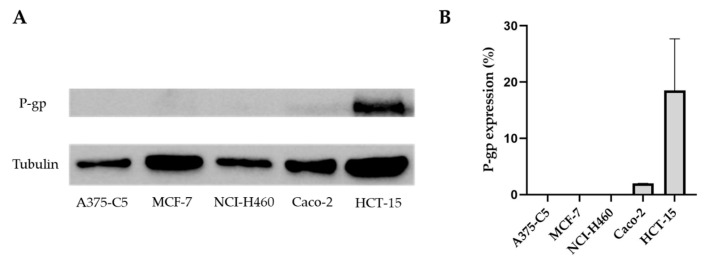
Western blotting assay to evaluate Pgp expression in the tumor cell lines A375-C5, MCF-7, NCI-H460, Caco-2, and HCT-15. Tubulin was used as a loading control. (**A**) Representative results of Pgp expression. (**B**) Pgp expression levels in the cell lines under study.

The GI_50_ of CDXs determined based on the cell lines A375-C5, MCF-,7 and NCI-H460 indicated considerable differences for the same compound, in the assayed cell lines. In this way and since Pgp is one of the main causes of drug resistance, the analysis of the expression of this protein in the assayed cell lines was performed. For this purpose, protein extraction from the different cell lines was carried out, and Pgp expression was evaluated via Western blotting. Despite the differences observed in GI_50_ values, no Pgp expression was observed in these cell lines. Given these results, new cell lines with possible Pgp expression were included in the assay, namely two colon adenocarcinoma cell lines, Caco-2 and HCT-15 (Figure 3). The results demonstrated that both cell lines express Pgp; however, the expression in the cell line HCT-15 was much higher. The enantiomeric pair **X1AELTrp** and **X1AEDTrp** were then tested on the HCT-15 cell line (Table S2, SM). On this cell line, GI_50_ values for these compounds were 20.0 ± 2.3 µM and 6.2 ± 1.4 µM, for **X1AELTrp** and **X1AEDTrp**, respectively, contrary to what happened with doxorubicin, where the GI_50_ value was 2.14 ± 0.15 µM; higher resistance to these compounds was not observed in the cell line expressing Pgp, compared to in the other cell lines, indicating that Pgp expression should not be the main mechanism of resistance to the compounds assayed.

### 2.3. Mechanism of Cell Death, Induced by the Most Promising CDXs, **X1AELTrp** and **X1AEDTrp**

**Figure 4:**
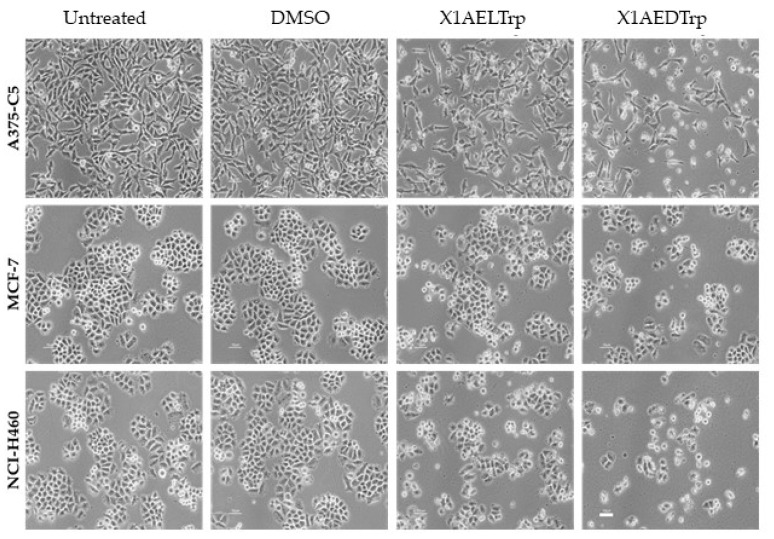
Representative phase-contrast microscopy images of untreated A375-C5, MCF-7, and NCI-H460 cells and cells treated with **X1AELTrp** and **X1AEDTrp** compounds, for 24 h. Cells treated with DMSO were used as controls. Scale bar, 10 µm.

**Figure 5:**
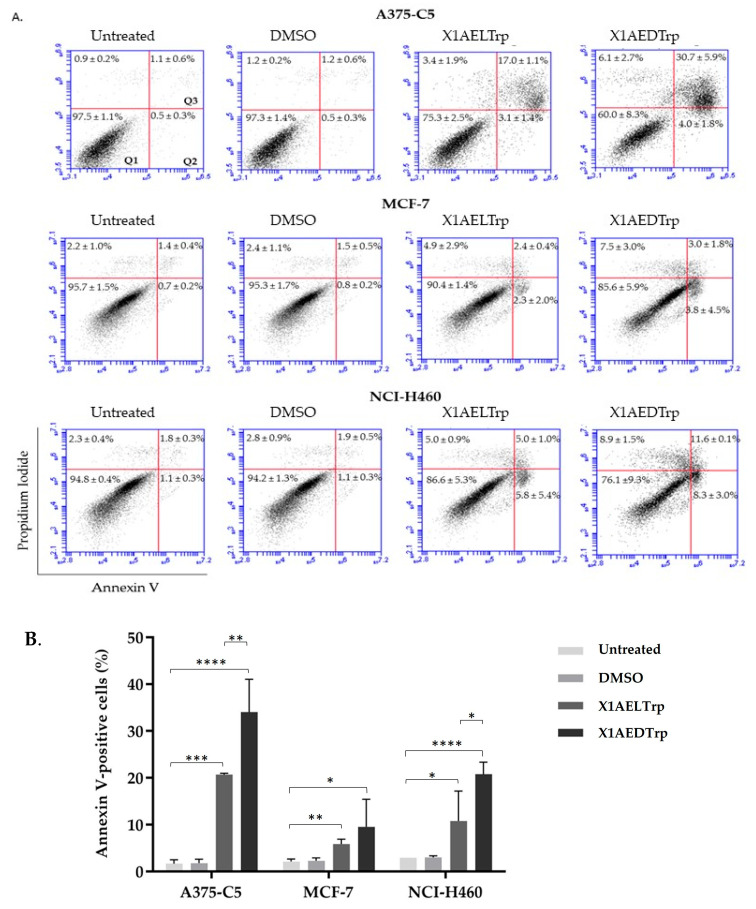
Cancer cells treated with **X1AELTrp** and **X1AEDTrp** undergo apoptotic cell death. (**A**) Representative flow cytometry histogram of Propidium iodide versus Annexin V-FITC intensity in untreated and **X1AELTrp**- and **X1AEDTrp**-treated cells, at 24 h. DMSO was used as a control. Q1 represents live cells (Annexin V-negative/PI-negative), Q2 corresponds to the early stage of apoptosis (Annexin V-positive/PI-negative), and Q3 denotes the late stage of apoptosis/secondary necrosis (Annexin V-positive/PI-positive [40,41]). (**B**) Quantification of AnnexinV-positive cells based on the data shown in (**A**). * *p* < 0.05, ** *p* < 0.01, *** *p* < 0.001, and **** *p* < 0.0001.

To gain insights into the cytotoxic mechanism by which **X1AELTrp** and **X1AEDTrp** exert their antitumor effect, the A375-C5, MCF-7, and NCI-H460 cancer cells were treated with these compounds for 24 h at concentrations corresponding to 2-fold the GI_50_ values previously obtained, and the cells were observed under phase contrast microscopy. Untreated and DMSO-treated cells were included as controls. The presence of wrinkled and detached cells was observed, which was more evident after **X1AEDTrp** treatment, suggesting cell death (Figure 4). Accordingly, cells were collected, and Annexin V/PI staining was performed and analyzed via flow cytometry to detect apoptosis (Figure 5). In untreated and DMSO-treated cells, the percentage of apoptosis was residual (<5% for all cell lines), as expected. In contrast, a significant increase in apoptotic cells was observed (Figure 4) after treatment with **X1AELTrp** and **X1AEDTrp**. As observed under microscopy, the percentage of apoptotic cells was higher after exposure to **X1AEDTrp** than with **X1AELTrp**. Overall, these results indicated that **X1AELTrp** and **X1AEDTrp** induce cancer cell death via apoptosis and that **X1AEDTrp** is more potent than its enantiomer **X1AELTrp**.

### 2.4. Alterations in Cancer Cell Metabolism, Induced by the Most Promising CDXs, **X1AELTrp** and **X1AEDTrp**

**Figure 6:**
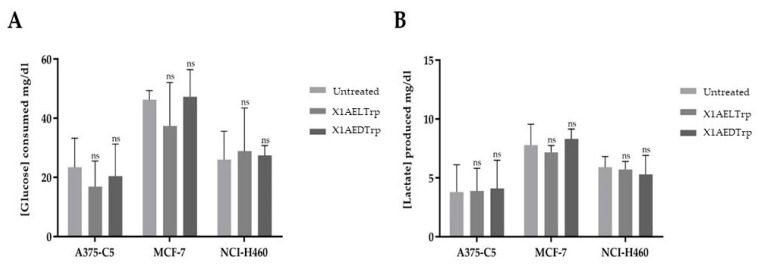
Concentration of glucose consumed and lactate produced by the three cell lines, A375-C5, MCF-7, and NCI-H460. (**A**) Concentrations of glucose consumed, in mg/dL, 48 h after cell treatment, for the different compounds, compared to untreated cells (control). (**B**) Concentrations of lactate produced by cells, in mg/dL, after treatment with the different compounds, compared to untreated cells (control). ns: statistically not significant.

Altered metabolism should be considered a core hallmark of cancer. In fact, tumor cells undergo metabolic reprogramming, with the ATP obtained by these cells mostly produced via aerobic glycolysis and not via oxidative phosphorylation, even in the presence of O_2_ (Warburg effect), leading to a high consumption of glucose and lactate production. This metabolic alteration in cancer cells can contribute to the increased proliferation and aggressiveness of the tumor. Thus, to verify whether the selected CDXs, **X1AELTrp** and **X1AEDTrp**, could target this hallmark and to further unravel their mechanism of action, extracellular glucose and lactate levels were quantified in all cancer cell lines assayed (A375-C5, MCF-7, NC-H460, and HCT-15), exposed to these compounds for 48 h, at a concentration corresponding to the respective GI_50_ for each cell line (Figure 6). No significant differences were obtained for all cell lines, comparing cells treated with these compounds with control (untreated) cells, indicating that their mechanism of action and cytotoxicity should be associated with other cancer characteristics.

### 2.5. Evaluation of the Functionality of the Multidrug Resistance Protein Pgp Based on a Yeast Strain Expressing the Human MDR1 Gene

**Figure 7:**
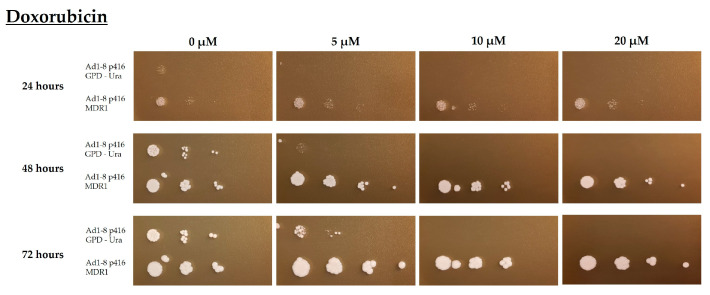
Growth of the *S. cerevisiae* strains Ad1-8 p416 GPD-Ura and Ad1-8 p416 MDR1 in YPD medium with different concentrations of doxorubicin, a known Pgp substrate (5 μM, 10 μM, and 20 μM), after 72 h of incubation at 30 °C. Untreated cells (0 μM) were used as a control.

As previously mentioned, Pgp is a transmembrane glycoprotein, encoded by the MDR1 gene, often involved in multidrug resistance phenotypes in tumor cells. To determine if the compounds assayed in this work could be possible Pgp substrates, a recombinant *Saccharomyces cerevisiae* yeast strain expressing the human MDR1 gene and its endogenous efflux pumps (AD1-8 p416 MDR1) deleted was used, as well as the respective control strain, transformed with an empty plasmid lacking the MDR1 gene (AD1-8 p416 GPD-Ura). The yeast *S. cerevisiae* is a unicellular organism often used as a host in heterologous expression systems of human genes [42]. First, the functionality of the protein in the humanized yeast was evaluated, using doxorubicin, a well-known Pgp substrate (Figure 7).

As expected, both strains, expressing or not expressing Pgp, grew in YPD medium without the compound, used as a control. However, when doxorubicin was added to the plates, only the strain transformed with the MDR1 gene presented growth; this difference was more visible at the highest concentrations, which confirms the functionality of the human gene MDR1 in the transformed strain. These results indicate that humanized yeast expressing the human gene MDR1 is a valid model to screen Pgp substrates, which can have valuable clinical interest in the screening of drugs.

### 2.6. Phenotypic Evaluation of the Growth of the Saccharomyces Cerevisiae Yeast Strains AD1-8MDR and AD1-8GPD in the Presence of Different Concentrations of the CDXs **X1AELTrp** and **X1AEDTrp**

**Figure 8:**
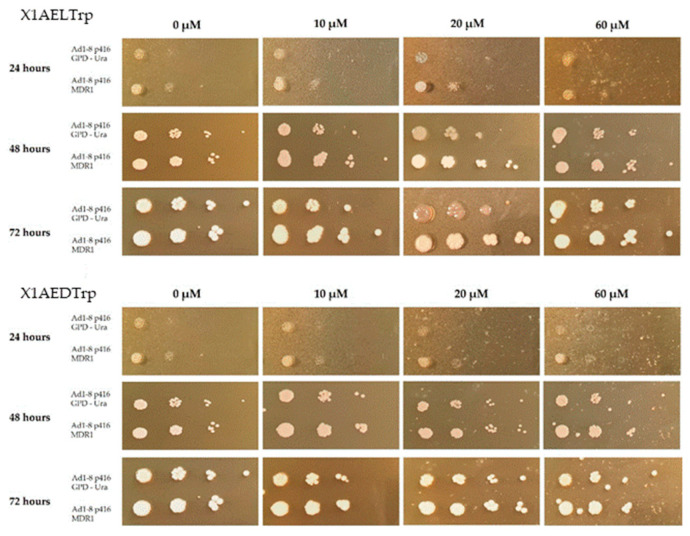
Growth of the *S. cerevisiae* strains Ad1-8 p416 GPD-Ura and Ad1-8 p416 MDR1 in YPD medium with different concentrations of **X1AELTrp** and **X1AEDTrp** (10 μM, 20 μM, and 60 μM), after 72 h of incubation at 30 °C. Untreated cells (0 μM) were used as a control.

After confirming the functionality of the human gene MDR1 in yeast, this model was used to find out if the compounds **X1AELTrp** and **X1AEDTrp** could also be Pgp substrates (Figure 8). In this analysis, the concentrations used were higher than those used with doxorubicin, taking into account the differences in the respective GI_50_ values and the effect of the compounds on yeast growth. It can be observed that for CDXs **X1AELTrp** and **X1AEDTrp**, both strains, either transformed with the empty plasmid or transformed with the MDR1 gene, presented growth in the presence of the compound, regardless of the concentrations of the compounds, and for the maximum time of exposure to the compounds (72 h). These observations indicate that these CDXs, in opposition to doxorubicin, are not toxic to yeasts and do not allow conclusions to be drawn as to whether they are or are not Pgp substrates. Different results, however, were obtained with other xanthones, which were not further exploited due to their high GI_50_, where it was verified that they are possibly Pgp substrates, although it was necessary to use high drug concentrations to see such an effect in Figure S1 (SM).

Although no significant differences in the GI_50_ were observed in cells expressing or not expressing Pgp, this does not exclude the role of Pgp in the resistance to the compounds assayed. In fact, the cell lines have different genetic backgrounds and provenience, and other factors can be involved in mechanisms of resistance. The yeast *Saccharomyces cerevisiae* is a unicellular eukaryotic organism, well studied at the genetic and physiological levels, having been the first eukaryotic organism to have its genome completely sequenced [43]. Like bacterial systems, and unlike animal cells, the yeast *S. cerevisiae* is capable of growing in simple and inexpensive culture media, presenting short generation times, and reaching high cell densities. Being a eukaryotic organism, it shares several cellular processes with higher eukaryotic organisms, showing the ability to carry out post-translational modifications, similar to those that occur in the synthesis of functional proteins in these organisms [44]. The yeast *S. cerevisiae* has been manipulated to express heterologous genes, as it has extremely versatile metabolism, presents several genetic tools, and satisfies the biosafety norms for application in human beings. Thus, this microorganism was used to express Pgp, aiming to find a system suitable to screen drugs that can be identified as substrates of this protein. ABC transporters are found in the cells of all organisms, often in the plasma membrane. In yeast, there are two main families of efflux proteins consisting of several members: the Major Facilitator Superfamily (MFS) and the ABC transporter family, composed of six subfamilies and presenting a wider range of substrates [45,46]. Currently, more than 52 yeast strains are available in which the main efflux pumps involved in pleiotropic drug resistance (PDR) are deleted [46,47]. One of these examples is the AD1-8 strain of *S. cerevisiae*, used in this work for the expression of Pgp, in which different endogenous efflux pumps are deleted, as well as in the Pdr3 transcription factor, to constitute a suitable system for the expression of ABC proteins, as the expression of endogenous efflux pumps could influence the functional analysis [47]. It was verified that in the yeast expressing the MDR1 human gene, there was an increased resistance to doxorubicin, a known Pgp substrate, compared to that in the control strain, transformed with the empty plasmid, indicating that the gene was functional in this expression system. These results indicate that the yeast *S. cerevisiae* is a good model for the screening of Pgp substrates. Nevertheless, it has some limitations, as some drugs are toxic for humans but not for yeast, as was observed with some of the CDXs assayed, and so it could not be validated using this model. In this way, the in silico analysis can also contribute to a more complete study of the influence of Pgp on the anti-tumor activity of these derivatives.

### 2.7. Docking Scores of the CDXs and Positive Controls Based on the Drug-Binding Pocket of the Human Inward Pgp Model

**Figure 9:**
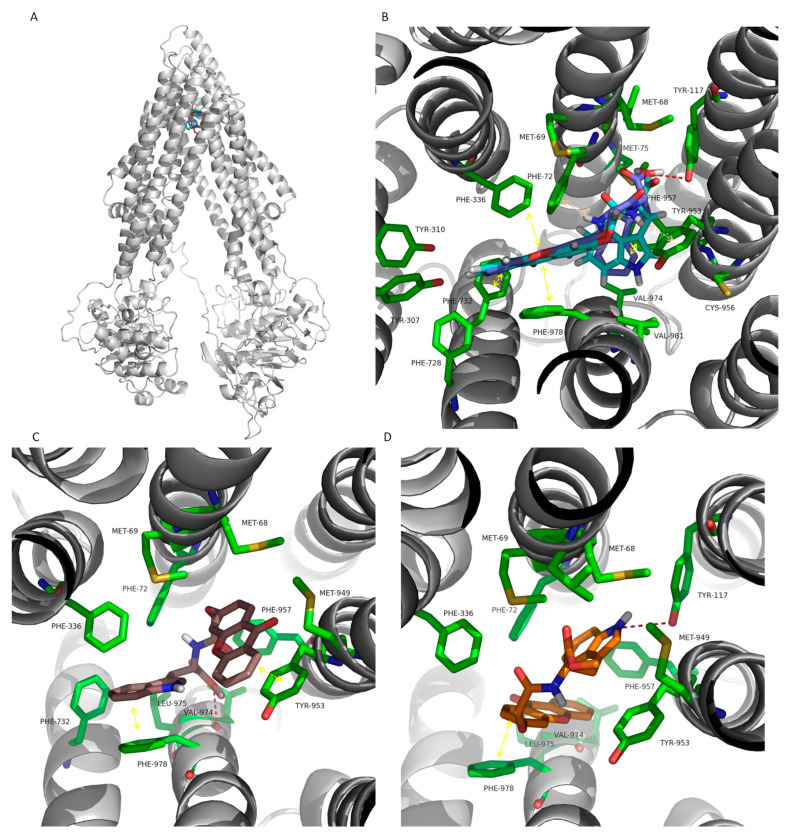
(**A**) Ribbon representation for the P-glycoprotein (Pgp) model and test molecules docked into the transmembrane domains (TMDs). (**B**) Detailed view of X1AADTrp (light blue sticks) and X1AADTrp (dark blue sticks), (**C**) **X1AELTrp** (brown sticks), and (**D**) **X1AEDTrp** (orange sticks) based on the drug-binding site on the interface of the TMDs. Hydrogen interactions, π-stacking interactions, and H-π interactions are represented as red broken lines, yellow double arrows, and orange doble arrows, respectively, and the involved residues are represented as green sticks that are labeled. The labeled residues without a representation of an interaction are involved in other types of interactions.

An extensive range of substrates that are structurally very diverse from each other can bind and be extruded by Pgp [48]. Consequently, although a compound may be predicted to be active by binding a target, it can be transported out of the cell by Pgp, thus reducing its intracellular concentration and its activity [49]. Pgp substrates are described as being lipophilic or amphiphilic, positively charged, and having a large size or molecular volume and electronegative groups [50,51]. Moreover, Pgp substrates generally have hydrogen bonding groups and p-electron rings that can establish hydrogen bonds, π-stacking, and cation-π interactions with the efflux pump [52]. Pgp substrates span several classes of drugs, such as anticancer drugs, antiepileptic, β-receptor and calcium channel blockers, steroids, opioids, immunosuppressants, HIV protease inhibitors, antiemetics, antimicrobials drugs, hypolipidemic agents, and antihistaminic [53]. Xanthones and thioxanthones have already been described as Pgp modulators [12,54]. The xanthonic scaffold’s chemical characteristics and different substitution patterns make them useful in the design of Pgp substrates, inhibitors, or inducers/activators [55,56]. Docking scores of the forty-six target structures and positive controls based on the drug-binding pocket of the human inward Pgp model confirmed that substrates present high affinity towards Pgp with a free energy of binding ranging from −6.5 to −12.7 kcal/mol (Table S3, SM). CDXs are predicted to bind to Pgp with energy within the positive control range, from −8 to −10.4 kcal/mol, and therefore are foreseen as having a high probability of interacting with Pgp. The binding site was located at the translocation pathway formed in the TMs interception [57] (Figure 9A). It has been suggested that steric complementarity and the strength of the interactions differentiate substrates from inhibitors, rather than distinct binding sites [16]. A example, some of the top scored CDXs, X1AADTrp, X1AALTrp, **X1AELTrp**, and **X1AEDTrp**, were more thoroughly analyzed (Figure 9B–D). Concerning X1AADTrp and X1AALTrp, a hydrogen interaction was established between the carboxylic acid group and Tyr-117. Moreover, parallel π-stacking interactions are established between the xanthone central ring and Phe-978 and between the indol group and Phe-957. Perpendicular π-π-stacking interactions are established between the xanthone ring and Pgp residues Phe-336 and Phe-732 and between the indol group and Phe-72. H-π interactions are also visualized between the xanthone -N-H and Tyr-953 (X1AADTrp) or Phe-72 (X1AALTrp) (Figure 9B). A hydrogen interaction was established between the **X1AELTrp** ester group and Val-974. This compounds also establish parallel π-stacking interactions with Phe-978 and Tyr-953 (Figure 9C). The **X1AEDTrp** indole group establishes a hydrogen interaction with Tyr-117, and the xanthonic ring system forms π-stacking interactions with Phe-978 (Figure 9D).

Phe-336, Phe-728, and Phe-978 have already been described, based on mutagenesis studies, as playing an important role in the recognition, binding, and transport of specific substrates by Pgp [58,59]. Furthermore, Phe732 has been described to interact with Pgp substrates based on molecular dynamics simulations [60], and Met-68, Tyr-307, Tyr-953, and Val-981 have been identified as important residues for substrate recognition based on molecular homology modeling and docking [57,61]. Recently, Met-68, Met-69, Tyr-310, Phe-336, Phe-728, and Tyr-953 have been found to be implied in the binding of the substrate vincristine to the Pgp structure obtained via cryo-EM [62].

Hence, the in vitro and silico studies further attest to the potential of xanthones to interact with Pgp, namely as substrates. The resistance mechanism is a complex process, where multiple interveners can be involved, with Pgp being one of the main players in this phenotype. The comparison of GI_50_s between cell lines expressing or not expressing Pgp is not totally suitable to analyze if putative antitumor drugs are or are not Pgp substrates, due to the complexity of the MDR phenotype and the multiplicity of mechanisms of resistance that can be present in the different cell lines. Here, we have demonstrated that either in silico studies, where the interaction between the compounds and Pgp can be analyzed, or the use of a “clean” system, like the yeast model presented here, where Pgp expression or its absence is the only factor that can affect yeast growth in the presence of the compound, can be used as a first approach to identify putative Pgp substrates. Nevertheless, some drawbacks can be present with the use of the yeast model, namely if the compounds are toxic to human cells, but not to yeast, as it was verified in this work for the CDXs **X1AELTrp** and **X1AEDTrp**. Furthermore, such findings should be confirmed afterward using human cells with Pgp assays. 

## 3. Materials and Methods

### 3.1. Chemicals

The commercially available reagents and solvents used in this work were purchased from Sigma Aldrich Co., Lisboa, Portugal, and were used without purification. In some assays, commercial doxorubicin, purchased from Sigma-Aldrich (Lisboa, Portugal), was used as a control. The stock solutions of CDXs and doxorubicin were reconstituted in sterile dimethyl sulfoxide (DMSO) Sigma-Aldrich, Lisboa, Portugal), which was then diluted in a fresh culture medium at the appropriate concentrations. All stock solutions were filtered and used immediately or stored at −20 °C.

The CDXs were previously synthesized according to the methodology described elsewhere [15]. Briefly, the 9-oxo-9*H*-xanthen-3-yl)oxy)acetic acid (100 mg, 0.37 mmol)—dissolved in dry tetrahydrofuran (20 mL) and then trimethylamine (100 µL, 0.72 mmol)—was added. The coupling reagent (1.2 eq. mmol) was added, and the solution was stirred for about 30 min. Afterward, the enantiomerically amino ester (1.7 eq.) was added, and the mixture was stirred at room temperature for 30 min up to 30 h. The solvent was evaporated, and the crude product was dissolved in dichloromethane and washed with a 5% HCl solution (2 *×* 13 mL), 5% NaHCO_3_ solution (2 *×* 15 mL), and water (3 *×* 25 mL). The organic layer was dried with anhydrous sodium sulfate and filtered, and the solvent was evaporated. The products were then crystallized to afford the CDXs of amino esters. The synthesized CDXs of the amino ester (50 mg) were hydrolyzed in methanol (10 mL) with NaOH 0.25 M (3.75 mL) at room temperature, and then, the solvent was evaporated, 10 mL of water was added, and it was acidified with concentrated HCl. The solid was collected via filtration under reduced pressure, washed with cold water, and recrystallized to afford the CDX of amino acids.

### 3.2. Cell Culture

Five human tumor cell lines, obtained from the European Collection of Cell Culture, were used in this study, namely A375-C5 derived from melanoma, MCF-7 derived from breast adenocarcinoma, NCI-H460 derived from non-small cell lung adenocarcinoma, and HCT-15 and Caco-2 derived from colorectal carcinoma. The HPAEpic cell line (Human Pulmonary Alveolar Epithelial Cells) was also included in the screening assays and was obtained from ScienCell Research Laboratories. All cell lines were maintained at 37 °C in a 5% CO_2_ humidified incubator, and all experiments were performed when exponentially growing cells presented more than 95% viability. A375-C5, MCF-7, and NCI-H460 were grown in RPMI-1640 culture medium (Roswell Park Memorial Institute, Biochrom, Cambridge, UK), supplemented with 5% heat-inactivated FBS (fetal bovine serum, Biochrom) and 1% of penicillin/streptomycin (Sigma-Aldrich, Lisboa, Portugal). HCT-15 cells were also cultured in the same medium but supplemented with 10% FBS. HPAEpic cells were cultivated in Dulbecco’s Modified Eagle Medium (DMEM, Lonza, Walkersville, MO, USA), supplemented with 10% FBS, 1% Non-Essential Amino Acids (NEAAs, Sigma-Aldrich, Lisboa, Portugal), and 1% penicillin/streptomycin. 

### 3.3. Cell Viability Assays

For the screening assays with CDXs, the sulforhodamine B (SRB) assay was performed to evaluate their effect on the cell viability of the different human cell lines. For that and for all cell lines, cells were seeded in 96-well plates (at a cell density of 5 × 10^3^ cells per well for cancer cell lines and 0.65 × 10^6^ cells per well for the HPAEpic cell line) and incubated overnight at 37 °C with 5% CO_2_. After this time, when the cells had adhered, the medium was removed and, after cell washing with PBS, replaced with medium containing the compounds at the desired concentrations. For each compound concentration, triplicates were used, as well as duplicates of the respective blank (wells containing medium without cells, but with the respective compound concentration). Untreated cells were used as controls and were taken as having 100% viability. To analyze if DMSO could affect cell viability, DMSO-treated cells with the drug volume replaced by the same amount of DMSO were used. After 48 h of incubation, the medium was removed, and the plates were fixed via incubation for 1 h at 4 °C with 50 µL of 50% TCA. The cells were then washed 5 times with deionized water, dried at room temperature, and stained for 30 min at room temperature with 50 µL SRB at 0.4% in acetic acid (*w*/*v*) (PanReac AppliChem ITW Companies, Darmstadt, Germany). After staining, the cells were rinsed 5 times with 1% (*v*/*v*) acetic acid and after drying at room temperature, the cell–SRB complex was solubilized in 100 µL of 10 mM Tris per well, and the absorbance was read at 515 nm using a plate reader (Synergy 2, Biotek, Winooski, VT, USA). In all the SRB assays performed, an additional plate was prepared in the same way as described but fixed at the time of the addition of the compounds to calculate the cell density in the plates at the time of the addition of the compounds (designated as Plate T0).

The concentration of each compound that caused 50% inhibition of cell growth (GI_50_) was determined for the different cell lines, based on the dose–response curve, using an automated Excel sheet. 

### 3.4. Annexin V/Propidium Iodide Staining

A total of 0.12 × 10^6^ tumor cells were seeded with complete culture medium in a 6-well plate, allowing them to attach for 24 h. Then, cells were treated with the compounds to be assayed or with DMSO up to 0.25%, as a control for the compound solvent. Untreated cells were also included. Twenty-four hours later, for apoptosis detection, floating and adherent cells were harvested and further processed with the “Annexin V-FITC Apoptosis Detection Kit” (eBioscience, Vienna, Austria) for flow cytometry, according to the manufacturer’s instructions. Briefly, cells were suspended in 195 µL of binding buffer, followed by the addition of 5 µL of Annexin V-FITC. After an incubation of 10 min, while protected from light, cells were washed and resuspended in 190 µL of binding buffer, and 10 µL of Propidium iodide (PI, 20 μg/mL) was added. At least 20,000 events per sample were collected, and the data were analyzed with BD AccuriTM C6 Plus software, version 1.0.27.1. 

### 3.5. Image Processing

For phase-contrast microscopy images, a Nikon TE 2000-U microscope (Nikon, Amsterdam, The Netherlands) with a 10× objective was used. The Nikon microscope was coupled to the DXM1200F digital camera with Nikon ACT-1 software version 2.63 (Melville, NY, USA).

### 3.6. Pgp Expression Evaluation

For protein extraction, cells were grown until confluence in 6-well plates. The medium was then removed, and the cells were washed with PBS and ice-incubated for 20 min in lysis buffer (50 mM Tris HCl pH 7.5; 30 mM NaCl; 0.5% Triton X-100 (*v*/*v*); 1 mM EDTA.Na; a cocktail of protease inhibitors). The suspension was transferred to an ice-cooled Eppendorf and centrifuged for 5 min at 13,000 rpm and 4 °C (pre-refrigerated centrifuge). The supernatant was collected and stored at −80 °C until use. The proteins were quantified using the “BCA Protein Assay Kit” (Pierce^TM^ BCA Protein Assay Kit, Thermo Scientific, Waltham, MA, USA), following the instructions provided by the manufacturer, using bovine serum albumin as the standard. 

Pgp expression in cancer cell lines was analyzed via Western blotting, according to standard procedures. Briefly, 20 µg of protein was separated via sodium dodecyl sulfate polyacrylamide gel electrophoresis (SDS–PAGE), on a 7.5% polyacrylamide separating gel, and transferred to a nitrocellulose membrane (Trans-Blot Turbo Blotting System, Bio-Rad, Hercules, CA, USA). For blocking, the membranes were incubated for 1 h at room temperature with 5% non-fat dried milk solubilized in TBST (10 mM Tris/HCl, pH 7.5, 150 mM NaCl, and 0.2% Tween 20). The membranes were then incubated with a Pgp antibody (rabbit anti-Pgp 1:1000, Abcam, Cambridge, UK) in a shaking bath at 4 °C overnight. After incubation, the membranes were again washed in a 1% non-fat milk solution in TBST and then incubated with a secondary antibody coupled to horseradish peroxidase (anti-rabbit IgG 1:1500, SIGMA) for one hour at room temperature. After incubation, the membranes were washed 3 times with Tris-Buffered Saline (TBS) buffer for 10 min at room temperature, with gentle agitation. As a loading control, an α-Tubulin (diluted 1:200, Abcam) antibody was used. Bands were visualized using enhanced chemiluminescence (ECL), and the protein content of the visualized bands was determined using ImageLab software (version 6.1.0, Bio-Rad), after measuring the density of each band and normalizing against the respective α-Tubulin density.

### 3.7. Metabolic Assays: Determination of Extracellular Levels of Glucose and Lactate

To evaluate the effect of the selected compounds on cancer cell metabolism, glucose and lactate levels in the extracellular medium were measured. Cells were incubated in 96-well plates at a density of 0.1 × 10^3^ cells/well. After this time, cells were treated with the respective IC_50_ of each compound for 48 h. Aliquots of 2 μL of the culture medium were used, and the metabolites were quantified using commercial kits (Spinreact, Sant Esteve de Bas, Girona, Spain), according to the manufacturer’s protocols. This analysis was performed in triplicate for each compound and concentration. The results presented correspond to the average of three independent experiments. For the statistical analysis, GraphPad Prism 9 software was used with ANOVA.

### 3.8. Yeast Strains and Media

Two *Saccharomyces cerevisiae* strains derived from the AD1-8 strain (MATα, pdr1-3, ura3, his1, yor1Δ::hisG, snq2Δ::hisG, pdr5-Δ2::hisG, pdr10Δ::hisG, pdr11Δ::hisG, ycf1Δ::hisG, pdr3-Δ2::hisG, pdr15Δ::hisG), kindly provided by André Goffeau [46], deleted in endogenous efflux pumps, were used: the AD1-8-p416-MDR1 strain, transformed with plasmid p416-GPD-URA containing the MDR1 gene, and the AD1 strain AD1-8–p416-GPD-Ura, harboring the empty plasmid. These yeast strains were previously constructed in our lab, via the transformation of the AD1-8 strain with the centromeric expression plasmid p416-GPD, containing the constitutive promoter GPD (Glyceraldehyde 3-Phosphate Dehydrogenase) [63] (Figure S2) and the MDR1-expressing plasmid p416-GPD-MDR1, also previously constructed in our lab, derived from plasmid p416-GPD via insertion of the MDR1 gene into the EcoRI and SalI restriction sites of the multicloning site, using the Gap Repair method (unpublished results). Yeast strains were maintained in 0.67% (*w*/*v*) yeast nitrogen base (YNB), 2% (*w*/*v*) glucose, and supplemented with the amino acids necessary for their prototrophic growth. Yeast Extract Peptone Dextrose medium (YPD), (1% (*w*/*v*) yeast extract, 1% (*w*/*v*) peptone, 2% (*w*/*v*) glucose) was also used for plate growth, supplemented, when appropriate, with different concentrations of the compound to be assayed. Agar (2.0% (*w*/*v*) was added for solid media. Both liquid and solid *S. cerevisiae* cultures were grown at 30 °C.

### 3.9. Evaluation of CDXs Effect on the Growth of S. cerevisiae Strains, Expressing or Not Expressing Pgp

To evaluate if the selected CDXs were possible Pgp substrates, the *S. cerevisiae* strains AD1-8-p416-MDR1 and AD1-8-p416-GPD, expressing or not expressing the protein, were grown in YPD solid medium supplemented with the compounds under study. For that, a suspension of cells from both strains at an OD of 0.1 at 600 nm was used. The suspensions were then diluted to 10^−1^, 10^−2^, 10^−3^, and 10^−4^, and 3 μL of each dilution was plated on plates containing the compound under study at different concentrations (5 μM, 10 μM, and 20 μM). As a control, the same cells were plated in YPD medium, without the compound. Cell growth was evaluated until 72 h, and the plates were photographed at this time. Plates supplemented with doxorubicin, a known Pgp substrate, were used as controls to ensure that the gene *MDR1* was functional in the yeast model.

### 3.10. Statistical Analysis

All assays were performed at least in duplicate from three independent experiments. Data were expressed as the mean ± standard deviation (SD), and statistical analysis was carried out in GraphPad Prism 8 (GraphPad Software Inc., San Diego, CA, USA) using an unpaired *t*-test.

### 3.11. Docking Studies

The structures of the forty-six CDXs and 20 known substrates (actinomycin D, aldosterone, calcein AM, colchicine, corticosterone, daunorubicin, dexamethasone, docetaxel, doxorubicin, endosulfan, etoposide, gefitinib, Hoechst-33342, imatinib, methylparathion, paclitaxel, rhodamine-123, topiramate, topotecan, and vincristine) [29] used as positive controls, were created and minimized using the Austin Model 1 (AM1) semi-empirical quantum mechanics force field until the gradient between any two successive steps in the geometry search was less than 10^−1^ kcal/mol·Å^−2^, using Hyperchem 7.5 (Hypercube, Florida, USA) [64]. Molecules were prepared for docking using AutodockTools [65]. The in silico docking of test molecules and positive controls was performed based on a human Pgp model (based on mouse Pgp—pdb code 4Q9H) using AutoDock Vina (Molecular Graphics Lab, Berkeley, CA, USA) [66]. Model creation and validation are already published elsewhere [67]. The binding site was defined by a grid box with dimensions of 30 × 30 × 30 Å centered on the drug-binding pocket based on the translocation pathway. Default settings were employed in the simulations, and the top 9 poses were collected for each molecule. The lowest docking score value was associated with a more favorable binding conformation. PyMol 1.3 (Schrödinger, New York, NY, USA) [68] was used for visual inspection of results and graphical representations. 

## 4. Conclusions

Given the inhibitory activity on the growth of tumor cells, previously described for xanthone derivatives [12,13], as well as the rule of the stereochemistry based on the properties of these compounds [8,14,15], the cytotoxic activity of forty-six chiral derivatives of xanthones (CDXs), was evaluated using a panel of three human tumor cell lines, including A375-C5 derived from melanoma, MCF-7 derived from breast adenocarcinoma, and NCI-H460 derived from non-small cell lung adenocarcinoma. Most CDXs presented GI_50_ values higher than 150 µM. Nevertheless, some were found with GI_50_ values lower than 50 µM for at least two cell lines, namely **X1AELTrp** and **X1AEDTrp**, which presented GI_50_ values below 15 µM for all cell lines. 

The most evidence of enantioselectivity was found in that D enantiomer demonstrated a better cytotoxic power relative to its L enantiomer. 

Due to the promising results of **X1AELTrp** and **X1AEDTrp** for all assayed cell lines, further investigations were performed within this enantiomeric pair. As many drugs used in chemotherapy are Pgp substrates, the expression of Pgp was analyzed using the five cancer cell lines assayed. Only HCT-15 cells presented considerable expression of Pgp; however, the GI_50_ values of **X1AELTrp** and **X1AEDTrp** for this cell line were of the same order of magnitude, as determined for the other three. In opposition, when the non-tumor cell line HPAepiC was used, a selectivity index higher than 1 against cancer cells was found for both pairs, indicating that these compounds are considerably more specific to cancer cells, as desirable. It was also found that the main mechanism of death triggered by these compounds was apoptosis, and **X1AEDTrp** was more potent than its enantiomer **X1AELTrp**.

## Figures and Tables

**Table 1:** GI_50_ values of the chiral derivatives of xanthones (CDXs) with GI_50_ values < 50 µM. The cell lines used in this assay were A375-C5 (melanoma), MCF-7 (breast cancer), and NCI-H460 (non-small lung cancer).

	GI50 (µM)		
Compounds	A375-C5	MCF-7	NCI-H460
**X1AELTrp**	14.46 ± 2.10	11.61 ± 4.63	11.82 ± 3.97
**X1AEDTrp**	10.66 ± 4.32	13.52 ± 1.94	12.94 ± 2.12
**X1AELPG**	32.42 ± 0.25	40.08 ± 7.16	41.94 ± 1.04
**X1AEDPG**	37.16 ± 2.17	44.06 ± 0.56	46.95 ± 3.27
**X1AEDPA**	32.03 ± 5.64	44.43 ± 8.26	33.13 ± 6.72
**X1AEDT**	26.52 ± 8.71	>150	>150
**X1AED-Met**	28.05 ± 7.85	20.35 ± 4.6	>150
**X1AAD-Met**	>150	19.33 ± 7.75	42.1 ± 35.1
**X1AED-Val**	>150	>150	25.6 ± 7.19
**Doxorubicin**	0.41 ± 0.097	0.47 ± 0.22	0.35 ± 0.05

**Table 2:** Selectivity index of **X1AELTrp** and **X1AEDTrp** compounds.

	GI_50_ (μM) ^1^	Selectivity Index ^2^		
	HPAepiC	A375-C5	MCF-7	NCI-H460
**X1AELTrp**	40.90 ± 1.17	2.83	3.52	3.46
**X1AEDTrp**	43.58 ± 3.32	4.09	3.22	3.37
**Doxorubicin**	0.056 ± 0.013	0.14	0.12	0.16

^1^ The GI_50_ from the non-tumor HPAEpiC (human pulmonary alveolar epithelial cells from ScienCell Research Laboratories) cells was calculated as described above; ^2^ Selectivity Index: GI_50_ based on non-tumor cells/GI_50_ based on tumor cells. **Doxorubicin** was used as a positive control.

## Data Availability

The data presented in this study are available on request from the corresponding author.

## Supplementary Materials

The supporting information can be downloaded at: https://www.mdpi.com/article/10.3390/ijms25042121/s1.
