# Supplementary material for: Evaluation of Antitumor Activity of Xanthones Conjugated with Amino Acids
Source: Int J Mol Sci. 2024 Feb 9;25(4):2121. doi: 10.3390/ijms25042121 (PMC10889492; doi:10.3390/ijms25042121)
Supplement: Supplementary file 1 [file ijms-25-02121-s001.zip › ijms-2815808-supplementary.pdf]

Phenotypic evaluation of the growth of the Saccharomyces cerevisiae yeast strains AD1-8MDR and AD1-8GPD, in the presence of different concentrations of the CDXs X1AEPA-Met, X1AAD-Met and X1AED-Val

After confirming the functionality of the *MDR1* gene in the host strain of *Saccharomyces cerevisiae*, the growth of the strain YPD and YPD supplemented with the xanthones the CDXs X1AEPA, X1AAD-Met and X1AED-Val was evaluated, as described before. In the YPD plates containing different concentrations of the chiral xanthone derivatives (**Figure 1S**), the strain transformed with the empty plasmid (the restriction map of the plasmid is represented in **Figure 2S**) presented a lower growth compared to the strain hosting the MDR1 gene, with such differences being more noticeable for the highest concentration (90 μM). At lower concentrations (5 μM, 10 μM and 20 μM) of the compound, it was possible to observe growth, both in the strain transformed with the plasmid containing the MDR1 gene and in the empty plasmid, indicating that this compound is not toxic at these concentrations, for yeast cells, as observed for the xanthones X1AELTrp and X1AEDTrp. However, when the highest concentration was used, it was observed that there was increased cell growth in the strain transformed with the MDR1 gene. These results indicate that these compounds could be Pgp substrates, as the results have similarities with those obtained with doxorubicin, i.e., higher growth in cells transformed with the MDR1 gene, although in this case only in the presence of high concentrations of the compounds.

X1AEDPA

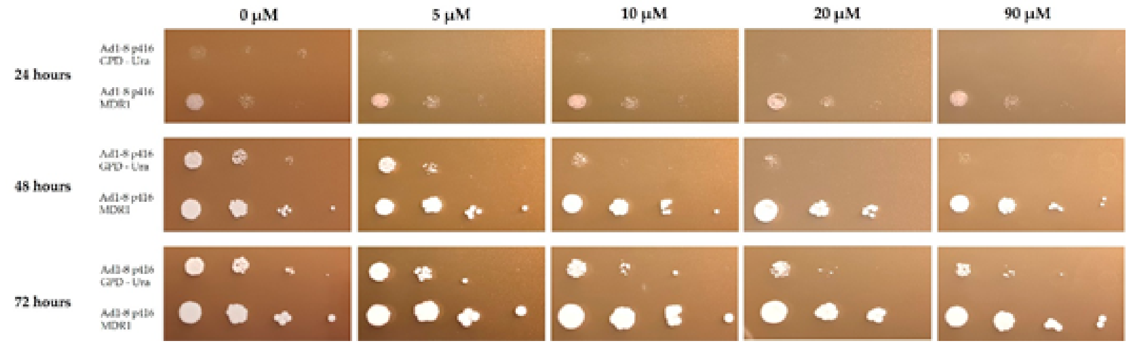

X1AAD-Met

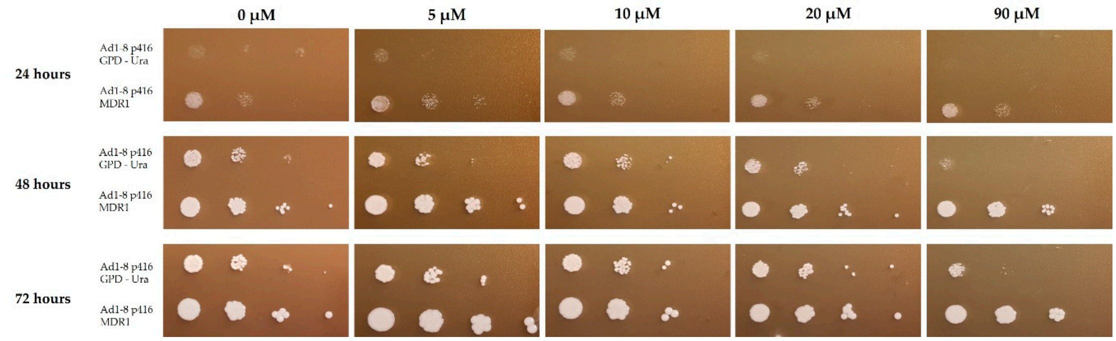

X1AED-Val

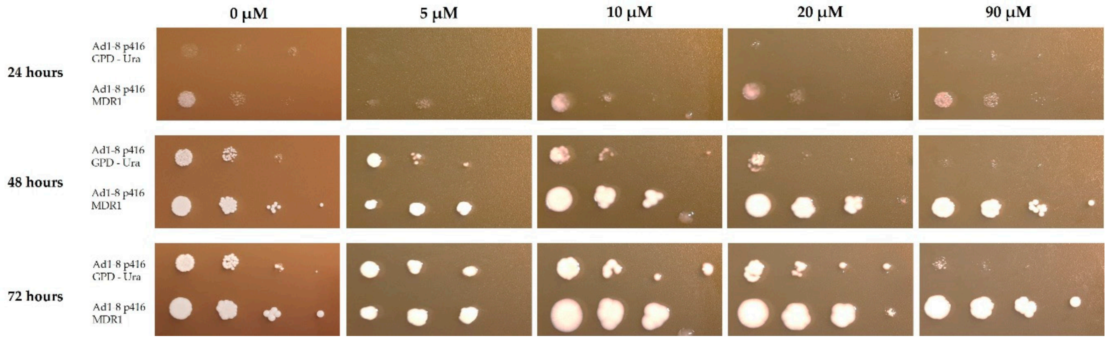

Figure S1. Growth of the *S. cerevisiae* strains Ad1-8 p416 GPD-Ura and Ad1-8 p416 MDR1 in YPD medium with different concentrations of X1AEDPA, X1AAD-Met and X1AED-Val (5 µM, 10 µM, 20 µM and 90 µM), after 72 hours of incubation at 30°C. Untreated cells (0 µM) were used as control.

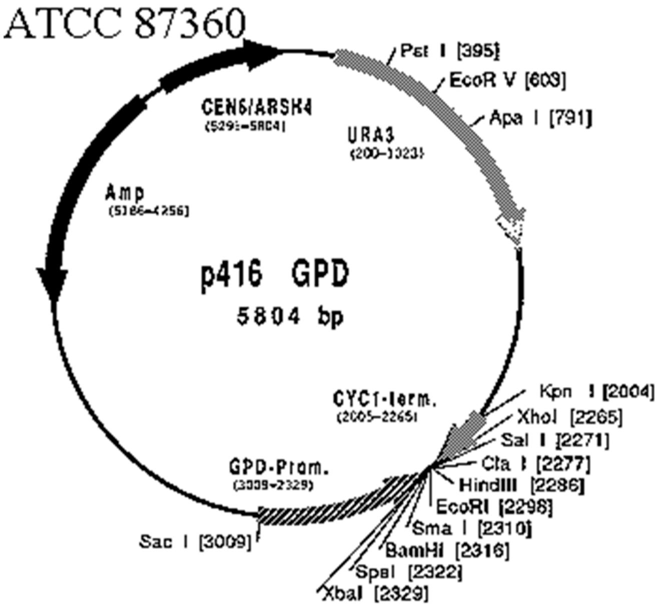

Figure S2. Restriction map of the centromeric expression plasmid p416-GPD, containing the constitutive promotor GPD (glyceraldehyde 3-phosphate dehydrogenase). MDR1 gene was inserted in this plasmid between the restriction sites EcoRI and SalI, by the Gap repair method.

Adapted from: https://www.atcc.org/products/87360

Table S1. Effect of chiral derivatives of xanthones (CDXs) in tumor cancer cells growth, evaluated by the respective GI_{50} value (in µM). The white lines correspond to the L enantiomer and the gray lines to the D one. The darker gray lines correspond to non chiral xanthones, conjugated with glycine. The cell lines used in this assay were A375-C5 (melanoma), MCF-7 (breast cancer), and NCI-H460 (non-small lung cancer). The amino acids used in conjugation were: Trp –Tryptophan, PG- phenylgycine, PA -

Phenylalanine, T- Tyrosine, Ser - Serine, Met - Methionine, Ala- Alanine, Pro - Proline, Val – Valine, Leu - Leucine, Thr – Threonine, Asp -Aspartic acid , Gly – Glycine.

**Compounds**	**GI_{50} (µM)**		
	**A375-C5**	**MCF-7**	**NCI-H460**
X1AELTrp	14.46 ± 2.10	11.61 ± 4.63	11.82 ± 3.97
X1AEDTrp	10.66 ± 4.32	13.52 ± 1.94	12.94 ± 2.12
X1AALTrp	>150	>150	>150
X1AADTrp	>150	>150	>150
X1AELPG	32.42 ± 0.25	40.08 ± 7.16	41.94 ± 1.04
X1AEDPG	37.16 ± 2.17	44.06 ± 0.56	46.95 ± 3.27
X1AALPG	>150	>150	>150
X1AAPG	>150	>150	>150
X1AELPA	68.67 ± 19.44	101.77 ± 20.68	66.4 ± 14.23
X1AEDPA	32.03 ± 5.64	44.43 ± 8.26	33.13 ± 6.72
X1AALPA	>150	>150	>150
X1AADPA	>150	>150	>150
X1AELT	>150	>150	>150
X1AEDT	26.52 ± 8.71	>150	>150
X1AALT	>150	>150	>150
X1AADT	73.5 ± 49.89	>150	>150
X1AEL-Ser	100 ± 29.09	>150	>150
X1AED-Ser	>150	>150	>150
X1AAL-Ser	>150	>150	>150
X1AAD-Ser	>150	>150	>150
X1AEL-Met	>150	>150	>150
X1AED-Met	28.05 ± 7.85	20.35 ± 4.6	>150
X1AAL-Met	>150	112.85 ± 24.94	106.08 ± 3.86
X1AAD-Met	>150	19.33 ± 7.75	42.1 ± 35.1
X1AEL-Ala	>150	>150	>150
X1AED-Ala	>150	>150	>150
X1AAL-Ala	>150	>150	>150
X1AAD-Ala	>150	122.33 ± 10.69	>150
X1AEL-Pro	>150	>150	>150
X1AED-Pro	133.47 ± 5.08	124.73 ± 4.77	102.87 ± 30.42
X1AAL-Pro	>150	>150	>150
X1AAD-Pro	>150	>150	>150

X1AEL-Val	>150	>150	95,73 ± 29,58
X1AED-Val	>150	>150	25.6 ± 7.19
X1AAL-Val	121.6 ± 12,27	>150	127.93 ± 14.53
X1AAD-Val	>150	>150	>150
X1AEL-Leu	>150	85.35 ± 3.04	>150
X1AED-Leu	>150	>150	>150
X1AAL-Leu	>150	>150	>150
X1AAD-Leu	>150	>150	59.2 ± 31.25
X1AAL-Thr	>150	>150	>150
X1AAD-Thr	>150	>150	>150
X1AEL-Asp	108.47 ± 9.61	>150	112 ± 19.03
X1AED-Asp	>150	>150	>150
X1AE-Gly	>150	>150	>150
X1AA-Gly	>150	>150	>150
Doxorubicin	0.41 ± 0.097	0.47 ± 0.22	0.35 ± 0.05

Table S2. Effect of chiral derivatives of xanthones (CDXs) and Doxorubicin in HCT15 cancer cells viability, evaluated by the respective GI_{50} value

Compound	GI_{50} (μM)			
	**A375-C5**	**MCF-7**	**NCI-H460**	**HCT-15**
X1AELTrp	14.46 ± 2.10	11.61 ± 4.63	11.82 ± 3.97	20.0 ± 2,3
X1AEDTrp	10.66 ± 4.32	13.52 ± 1.94	12.94 ± 2.12	6.2 ± 1,4
Doxorubicin	0.41 ± 0.097	0.47 ± 0.22	0.35 ± 0,05	2.14 ± 0,15

Table S3. Docking scores of test xanthones and positive controls on the drug-binding pocket of human inward P-gp model.

		**Docking scores (Kcal.mol^{-1})**
Test	X1AADTrp	-10.4
	X1AALTrp	-10.4
	X1AEDT	-10.2

	X1AELTrp	-10.1
	X1AADT	-10
	X1AADPA	-9.9
	X1AELPG	-9.9
	X1AALPA	-9.8
	X1AALT	-9.8
	X1AEDPA	-9.7
	X1AEDPG	-9.7
	X1AAD-Pro	-9.4
	X1AALPG	-9.4
	X1AAL-Pro	-9.4
	X1AEDTrp	-9.3
	X1AE-Gly	-9.3
	X1AADPG	-9.2
	X1AAL-Ala	-9.2
	X1AED-Pro	-9.2
	X1AEL-Pro	-9.2
	X1AAD-Ala	-9
	X1AAD-Val	-9
	X1AA-Gly	-9
	X1AAD-Leu	-8.9
	X1AAL-Ser	-8.9
	X1AAL-Val	-8.9
	X1AED-Ala	-8.8
	X1AEL-Ala	-8.8
	X1AAL-Leu	-8.7
	X1AAL-Thr	-8.7
	X1AED-Val	-8.7
	X1AEL-Val	-8.7
	X1AAD-Ser	-8.6
	X1AED-Asp	-8.6
	X1AED-Ser	-8.6
	X1AELPA	-8.6

	X1AEL-Asp	-8.5
	X1AEL-Ser	-8.5
	X1AAD-Thr	-8.4
	X1AED-Leu	-8.4
	X1AELT	-8.4
	X1AAL-Met	-8.3
	X1AAD-Met	-8.2
	X1AED-Met	-8.1
	X1AEL-Leu	-8.1
	X1AEL-Met	-8
**Positive controls**	**Actinomycin D**	-12.7
	**Imatinib**	-11.5
	**Hoechst33342**	-10.9
	**Rhodamine123**	-9.7
	**Aldosterone**	-9.4
	**Paclitaxel**	-9.4
	**Corticosterone**	-9.3
	**Vincristine**	-9.2
	**Docetaxel**	-9.2
	**Etoposide**	-9.1
	**Dexamethasone**	-9
	**Daunorubicin**	-8.9
	**Topotecan**	-8.8
	**Doxorubicin**	-8.6
	**Gefitinib**	-8.5
	**CalceinAM**	-8.1
	**Colchicine**	-7.8
	**Endosulfan**	-7.5
	**Topiramate**	-7.1
	**Methylparathion**	-6.5
